# Long Non-coding RNA Expression Profiling Identifies a Four-Long Non-coding RNA Prognostic Signature for Isocitrate Dehydrogenase Mutant Glioma

**DOI:** 10.3389/fneur.2020.573264

**Published:** 2020-11-20

**Authors:** Yusheng Chen, Yang Guo, Hang Chen, Fengjin Ma

**Affiliations:** ^1^Department of Neurosurgery, Henan Provincial People's Hospital, Zhengzhou, China; ^2^Department of Intensive Care Unit, The Third People's Hospital of Zhengzhou, Zhengzhou, China

**Keywords:** lncRNA profile, risk signature, IDH mutant, glioma, prognosis

## Abstract

**Background:** Isocitrate dehydrogenase (IDH) mutant is one of the most robust and important genetic aberrations in glioma. However, the underlying regulation mechanism of long non-coding RNA (lncRNA) in IDH mutant glioma has not been systematically portrayed.

**Methods:**In this work, 775 IDH mutant glioma samples with transcriptome data, including 167 samples from the Chinese Glioma Genome Atlas (CGGA) RNAseq dataset, 390 samples from The Cancer Genome Atlas (TCGA) dataset, 79 samples from GSE16011 dataset, and 139 samples from CGGA microarray dataset, were enrolled. R language and GraphPad Prism software were applied for the statistical analysis and graphical work.

**Results:** By comparing the differentially lncRNA genes between IDH mutant and IDH wild-type glioma samples, a four-lncRNA (JAG1, PVT1, H19, and HAR1A) signature was identified in IDH mutant glioma patients. The signature model was established based on the expression level and the regression coefficient of the four lncRNA genes. IDH mutant glioma samples could be successfully stratified into low-risk and high-risk groups in CGGA RNAseq, TCGA, GSE16011, and CGGA microarray databases. Meanwhile, multivariate Cox analysis showed that the four-lncRNA signature was an independent prognostic biomarker after adjusting for other clinicopathologic factors. Moreover, the Gene Ontology (GO) and Kyoto Encyclopedia of Genes and Genomes (KEGG) analyses revealed that the immune response and cellular metabolism were significantly associated with the four-lncRNA risk signature.

**Conclusion:** Taken together, the four-lncRNA risk signature was identified as a novel prognostic marker for IDH mutant glioma patients and may potentially lead to improvements in the lives of glioma patients.

The new established four-lncRNA risk signature was a prognostic factor for patients with IDH mutant glioma and may provide therapeutic targets in future.

## Introduction

Long non-coding RNAs (lncRNAs), which are initially regarded as spurious transcriptional noise, are recently reported to play roles in cellular development, cell metabolism, and posttranscriptional modification (1). The biological functions and specific expression patterns of lncRNA have given rise to novel viewpoints on a variety of diseases, including cancer (2). Many reports were proposed to investigate the molecular mechanism of lncRNA and its impact on cancer development. The lncRNA HOX Transcript Antisense RNA (HOTAIR), which regulates the transcriptional silencing of EZH2 and the epigenetic modification of H3K27, is reported as a negative prognostic factor in breast cancer and colon cancer (3, 4).

Isocitrate dehydrogenase (IDH) gene, encoded IDH enzyme, could catalyze the oxidative decarboxylation of isocitrate to α-ketoglutarate during the production of nicotinamide adenine dinucleotide phosphate (NADPH) (5). IDH mutation, one of the earliest detectable genetic alterations, has been identified as a favorable prognostic marker of glioma patients (6). Several clinical researches reported that IDH mutation was involved in the transcription and posttranslational modification (7, 8). However, the aberrant expressions of lncRNA in IDH mutant glioma remain unknown.

In this study, we investigated the change of lncRNA transcriptional profile and lncRNA-related biological function in IDH mutant gliomas. Moreover, a four-lncRNA risk signature was established as an independent prognostic factor for IDH mutant patients accurately and robustly. The findings in our study may help in understanding the features of the lncRNA in IDH-mutant glioma and potentially contribute to the clinical management of IDH mutant glioma patients.

## Materials and Methods

### Samples

The RNA sequence and microarray data and corresponding clinical data were downloaded from the Chinese Glioma Genome Atlas (CGGA) database (http://www.cgga.org.cn), The Cancer Genome Atlas (TCGA) database (http://cancergenome.nih.gov/), and the GSE16011 (https://www.ncbi.nlm.nih.gov/geo/query/acc.cgi?acc=gse16011). Four independent datasets were collected in our study, including CGGA RNAseq dataset (*n* = 167), TCGA RNAseq dataset (*n* = 390), GSE16011 dataset (*n* = 79), and CGGA microarray dataset (*n* = 139). Overall survival (OS) was calculated from the date of diagnosis until death or the end of follow-up. The clinicopathologic characteristics of the patients from three datasets are shown in Table 1.

**Table 1:** The clinical and molecular characteristics of glioma in three independent datasets.

	**CGGA RNAseq dataset**	**TCGA dataset**	**GSE16011**	**CGGA microarray dataset**
Age				
≤ 40 years	138	242	58	137
>40 years	187	385	156	164
Gender				
Male	203	363	149	180
Female	122	264	65	121
WHO grade				
Grade II	109	221	21	122
Grade III	72	245	66	51
Grade IV	144	161	127	128
IDH status				
Mutant	167	390	79	139
Wild type	158	237	136	162

*CGGA, Chinese Glioma Genome Atlas; TCGA, The Cancer Genome Atlas; IDH, isocitrate dehydrogenase*.

### Gene Ontology Analysis

Gene Ontology (GO) enrichment analysis, annotation, and visualization were performed using Metascape (http://metascape.org/) resource (9). A probability value of <0.05 was considered statistically significant.

### Statistical Analysis

Differentially expressed lncRNAs (DELs) between patients in IDH mutant group and IDH wild-type group were calculated with Limma R package. Associations between continuous variables were tested using Pearson correlation analysis. The survival distribution was estimated with Kaplan–Meier survival curves. Univariate Cox and multivariate Cox regression analyses were performed to identify independent prognostic factors. The risk-score formula for predicting survival was developed based on the expression level of lncRNA genes and the regression coefficient derived from the univariate Cox regression analysis. The four-lncRNA risk score for each patient was calculated as follows:

$$\begin{array}{ccc} \text{Risk score} & = & \left(\text{β}1\;\times \text{ expressio}{\text{n}}_{\left(\text{lncRNA}1\right)}\right)+\left(\text{β}2\;\times \text{ expressio}{\text{n}}_{\left(\text{lncRNA}2\right)}\right)\\ + & \left(\text{β}3\;\times \text{ expressio}{\text{n}}_{\left(\text{lncRNA}3\right)}\right)+\left(\text{β}4\;\times \text{ expressio}{\text{n}}_{\left(\text{lncRNA}4\right)}\right) \end{array}$$

Patients were separated into a low-risk group and a high-risk group based on the cutoff point (median value). The Venn diagram was generated by VennDiagram R package. Heatmap was conducted by pheatmap R package. The volcano plot was plotted by ggplot2 R package. Kaplan–Meier survival curves were conducted by GraphPad Prism (version 7.0). All statistical computations were performed with the statistical software environment R (version 3.6.0) (http://www.r-project.org/) and GraphPad Prism (version 7.0). *p* < 0.05 was considered statistically significant.

## Results

### Whole Genome Long Non-coding RNA Profiling in Isocitrate Dehydrogenase Mutant Glioma

**Figure 1:**
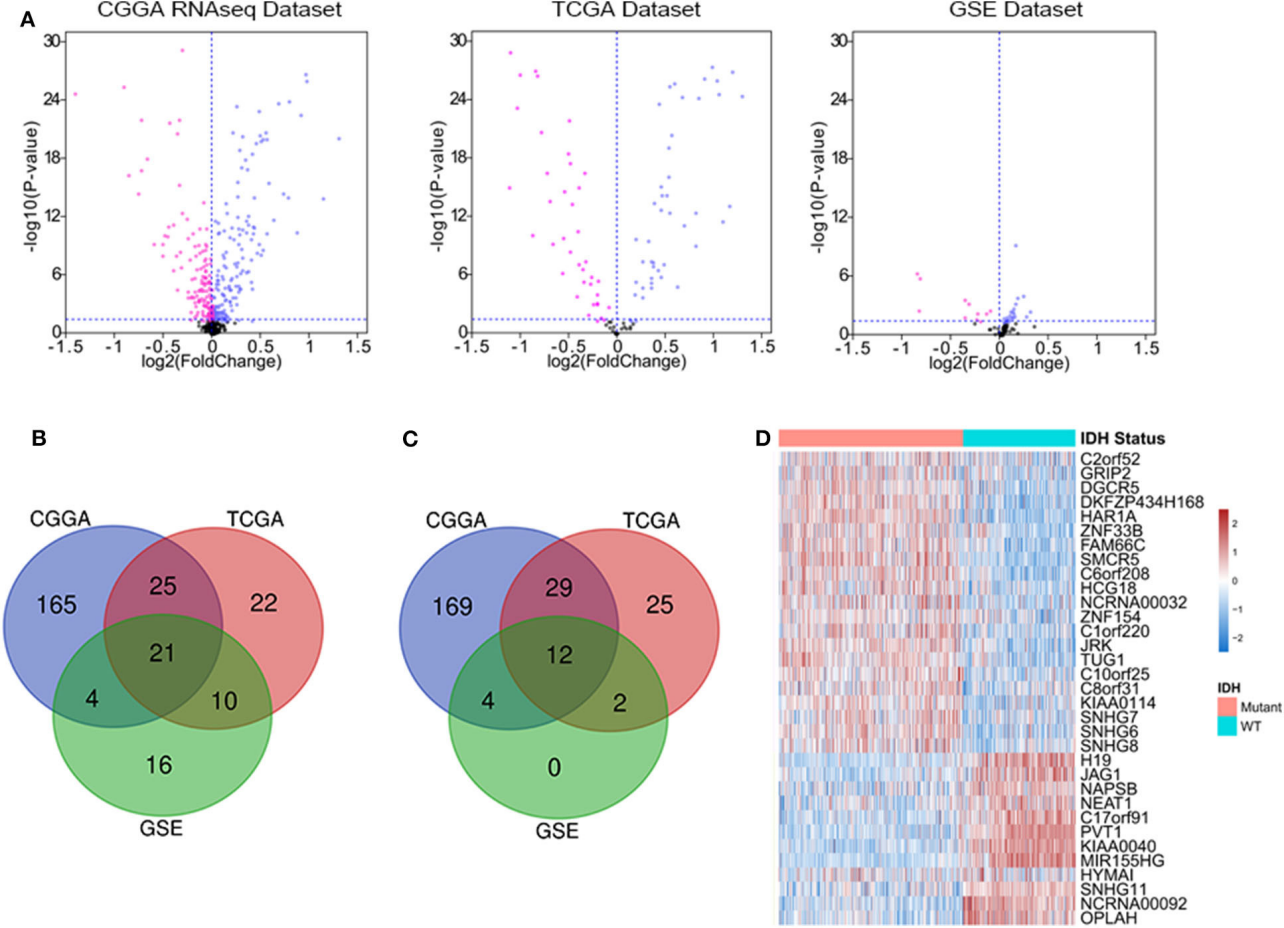
The deferentially expressed long non-coding RNA (lncRNA) genes that are significantly expressed between isocitrate dehydrogenase (IDH) mutant and IDH wild-type glioma. Volcano plots show the differentially expressed lncRNA genes in Chinese Glioma Genome Atlas (CGGA) RNAseq, The Cancer Genome Atlas (TCGA), and GSE16011 datasets. Each point represents a different gene, and the red and purple colors of the points categorize the downregulated and upregulated genes, respectively **(A)**. Venn diagram presents the workflow used to identify common upregulated and downregulated lncRNA genes in three datasets **(B,C)**. Expression patterns of 33 differentially expressed lncRNA genes in IDH mutant and IDH wild-type glioma **(D)**.

We downloaded the list of 56,946 lncRNA genes from LNCipedia database (https://lncipedia.org/) and identified the DELs between IDH1 mutant and IDH1 wild-type samples. The lncRNAs with a minimum of 2-fold difference in expression level and false discovery rate (FDR) of <0.05 were considered to be statistically significant. As shown in Figure 1A, volcano plots were generated to visualize the DELs between IDH mutant and IDH wild-type glioma in CGGA RNAseq, TCGA, and GSE16011 datasets. Red and purple plots represented the downregulated and upregulated lncRNAs, respectively. In total, 215 DELs, 78 DELs, and 51 DELs were significantly upregulated in IDH mutant glioma in CGGA, TCGA, and GSE16011 datasets, respectively (Figure 1B). Meanwhile, 214 DELs, 68 DELs, and 18 DELs were significantly downregulated in IDH mutant glioma in CGGA, TCGA, and GSE16011, respectively (Figure 1C). A total of 33 lncRNAs were found to be differentially expressed in all three independent datasets, including 21 lncRNA genes that were upregulated and 12 lncRNA genes that were downregulated in IDH1 mutant glioma (Figure 1D). The list of DELs is shown in Supplementary Table 1.

### Identification of the Four-lncRNA Signature for Isocitrate Dehydrogenase Mutant Glioma

**Figure 2:**
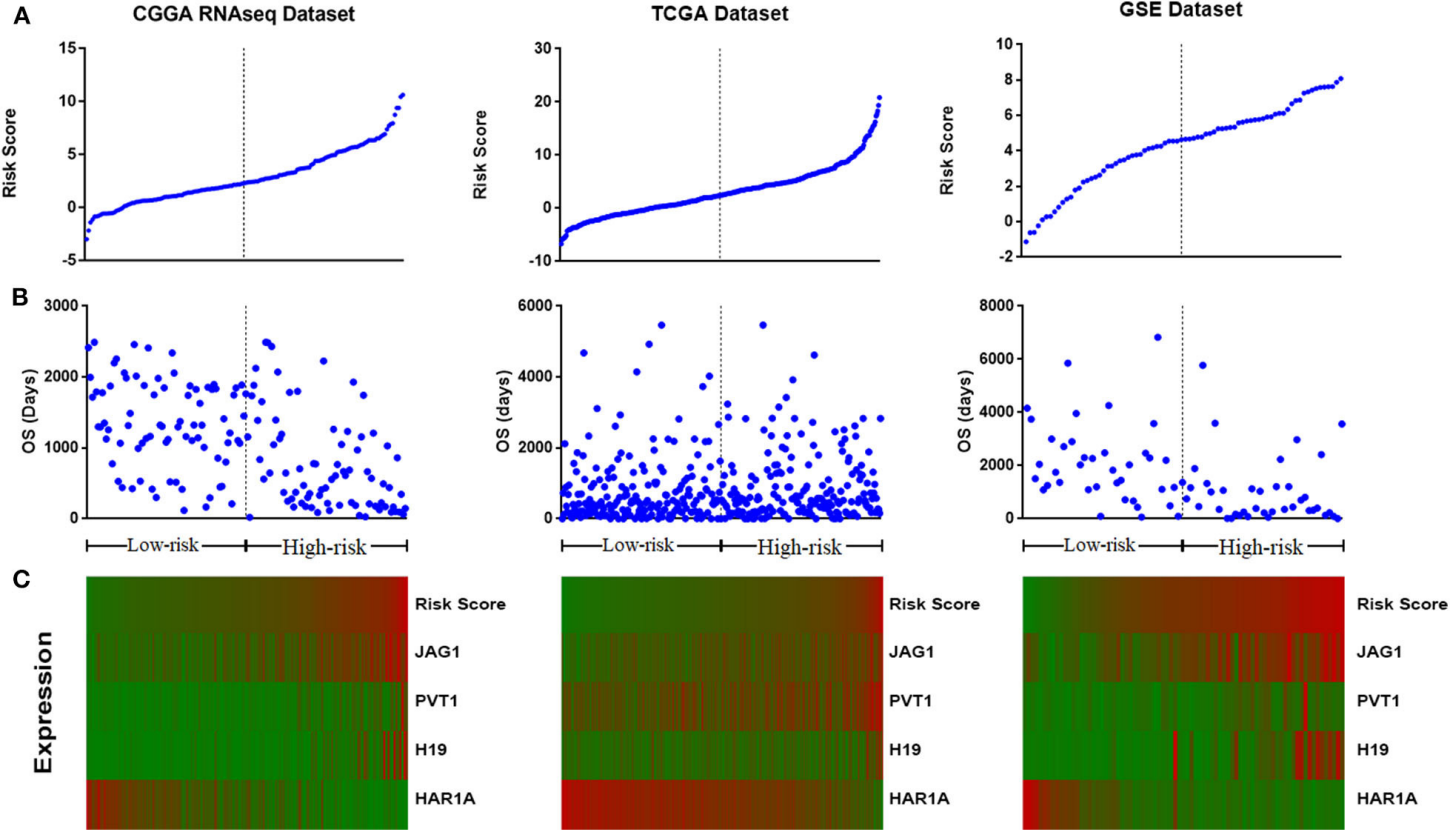
Distribution of risk score **(A)**, overall survival (OS) **(B)**, and the expression profile of four long non-coding RNA (lncRNA) genes with prognostic value **(C)**.

We assessed the prognostic performance of 33 DELs in IDH mutant glioma by univariate Cox regression analysis. The DELs were separated into risky lncRNAs [hazard ratio (HR) > 1] group and protective lncRNAs (HR < 1) group. With this definition, we found three risky lncRNAs (JAG1, PVT1, and H19) and one protective lncRNA (HAR1A) in all three databases. We then used four identified lncRNAs to construct a signature by risk score method. The risk score was calculated for each sample in three datasets. And then the IDH mutant patients were divided into the low-risk group and high-risk group based on the cutoff value (median four-lncRNA risk score). The four-lncRNA risk score and OS distribution are also shown in Figures 2A–C.

### Validation of Four-lncRNA Signature in Four Datasets

**Figure 3:**
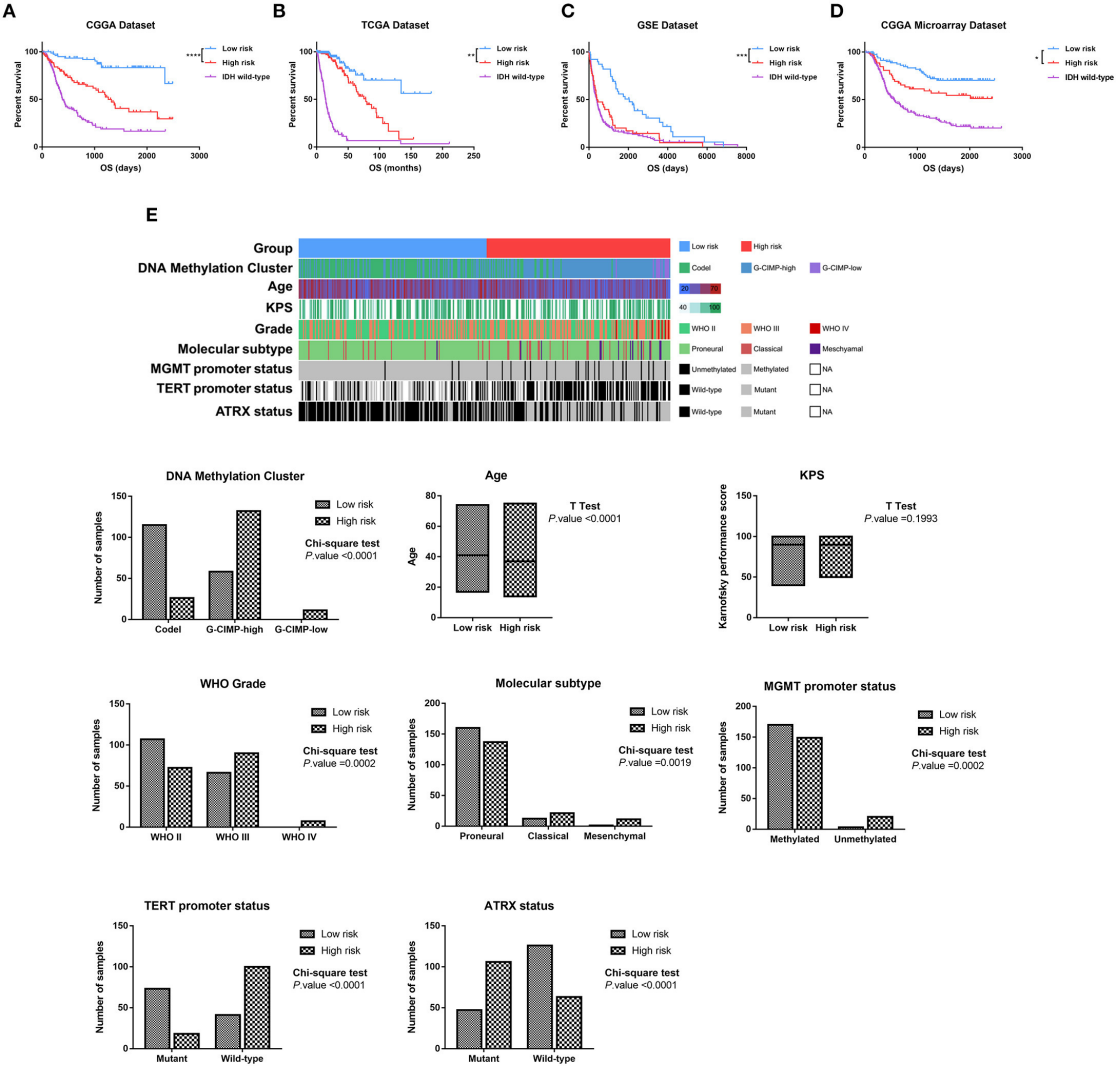
Based on the value of four-long non-coding RNA (lncRNA) risk signature, the isocitrate dehydrogenase (IDH) mutant glioma samples were divided into the low-risk and high-risk groups with different prognoses in Chinese Glioma Genome Atlas (CGGA) RNAseq **(A)**, The Cancer Genome Atlas (TCGA) **(B)**, GSE16011 **(C)**, and CGGA microarray datasets **(D)**. **(E)** Clinicopathologic features in different groups stratified by the signature in TCGA dataset. Rows represent corresponding items (DNA methylation cluster, age, KPS, grade, TCGA molecular subtype, MGMT promoter status, TERT promoter status, and ATRX status). *p < 0.05, **p < 0.01, ***p < 0.001 and ****p < 0.0001, respectively.

To evaluate whether the four-lncRNA risk signature could stratify glioma patients according to prognostic outcome, IDH mutant glioma samples in CGGA RNAseq dataset were divided into low-risk and high-risk groups according to the median risk score. Survival curves revealed that patients in the low-risk group had a significantly more favorable outcome than those in the high-risk group (Figure 3A, *p* < 0.0001). Meanwhile, the patients in the high-risk group had survival advantage over those in IDH wild-type group (Figure 3A, *p* = 0.0007). Then we evaluated the prognostic value of four-lncRNA risk signature in TCGA and GSE16011 datasets. As expected, the patients in the low-risk group had statically significantly longer OS than those in the high-risk group in TCGA (Figure 3B, *p* = 0.0013) and GSE16011 (Figure 3C, *p* < 0.0001) datasets. The CGGA microarray dataset, an independent validation dataset, was used to assess the prognostic value of four-lncRNA signature further. Similarly, the survival curves indicated that OS of high-risk patients was significantly shorter compared with that in the low-risk group (Figure 3D, *p* = 0.027). In addition, samples in IDH wild type displayed significantly shorter OS than those in the high-risk group in TCGA and CGGA microarray datasets (Figures 3B,D, *p* < 0.0001). However, the survival was similar in high-risk and IDH wild-type samples in GSE16011 dataset (Figure 3C, *p* = 0.4269).

Then we combined the methylation data, whole genome sequencing data, and clinical data to explore the features of IDH mutant glioma patients in the low-risk group and high-risk group. As shown in Figure 3E, the sample clusters, including codel DNA methylation cluster, the WHO grade II cluster, proneural cluster, MGMT promoter methylated cluster, TERT promoter mutant cluster, and ATRX wild-type cluster, were highly enriched in the low-risk group, whereas those in the high-risk group were moderately enriched for G-CIMP-high and G-CIMP-low clusters, WHO grade III and IV clusters, classical and mesenchymal clusters, MGMT promoter unmethylated cluster, TERT promoter wild-type cluster, and ATRX mutant cluster (*p* < 0.05, chi-square test). To our surprise, the patients in the high-risk group were significantly younger than in the low-risk group (*p* < 0.001, unpaired *t*-test).

These findings confirmed a strong correlation between the four-lncRNA risk signature and malignancy in glioma and indicated the powerful ability of the signature in stratifying the IDH mutant glioma.

### The Prognostic Value of Four-Long Non-coding RNA in Different Subtype of Isocitrate Dehydrogenase Mutant Glioma

**Figure 4:**
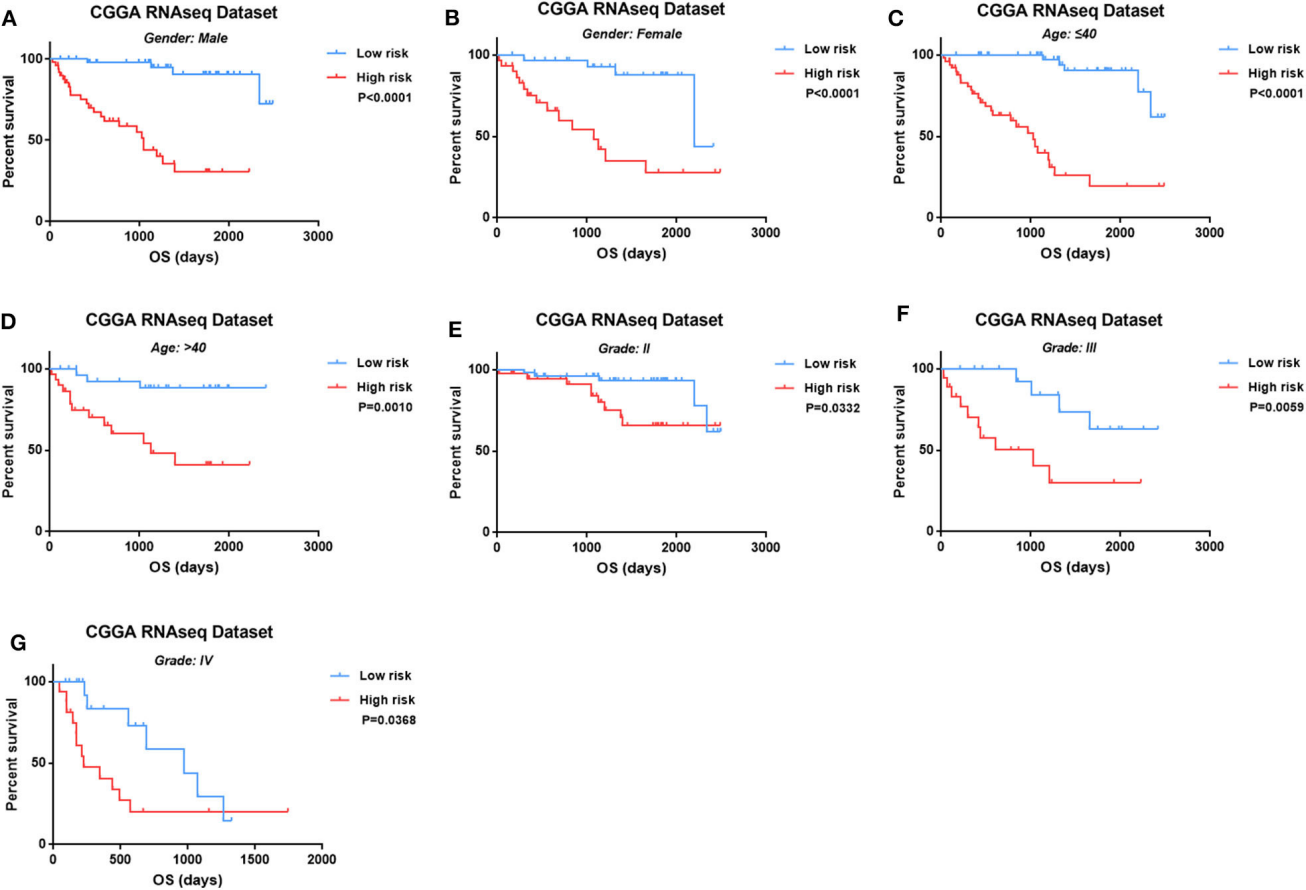
The four-long non-coding RNA (lncRNA) risk signature retained its prognostic significance in the subgroups stratified by gender **(A,B)**, age **(C,D)**, and grades **(E–G)**.

Then we observed the prognostic value of four-lncRNA risk signature in the different subtypes of glioma. We demonstrated the prognostic value of four-lncRNA signature in both male (Figure 4A, *p* < 0.0001) and female (Figure 4B, *p* < 0.0001). In addition, similar trends were observed in younger patients (Figure 4C, *p* < 0.0001) or older (Figure 4D, *p* = 0.001) patients. Moreover, we also found that an obvious difference in survival outcome existed between low-risk patients and high-risk patients in grade II (Figure 4E, *p* = 0.0332), grade III (Figure 4F, *p* = 0.0059), and grade IV (Figure 4G, *p* = 0.0368), respectively. Those results indicated that the prediction power of four-lncRNA risk signature was not decreased in the subgroup analysis for gender, age, and grade.

### The Four-Long Non-coding RNA Signature Is an Independent Factor for Isocitrate Dehydrogenase Mutant Glioma

**Figure 5:**
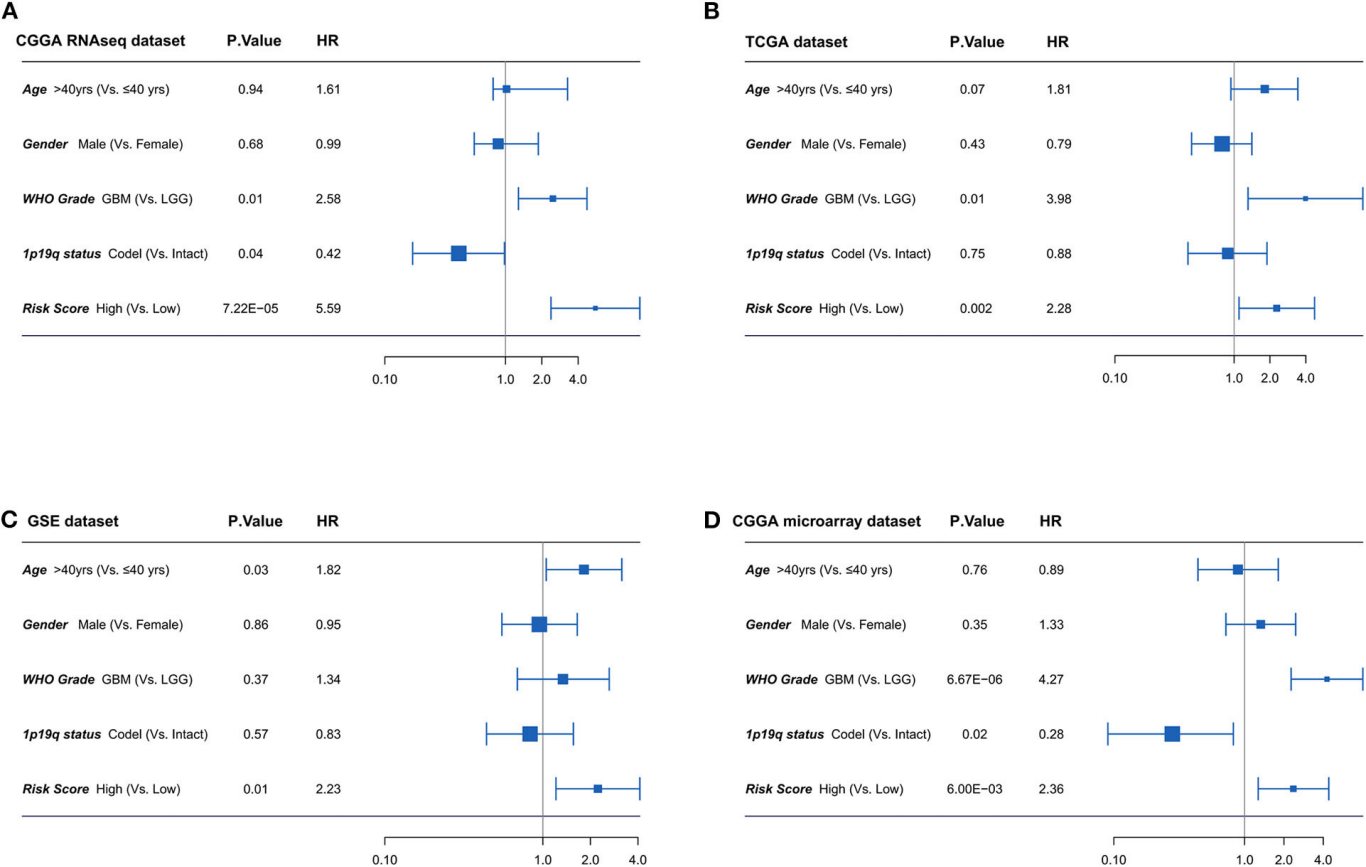
**(A-D)** Forest plot of hazard ratios for overall survival assessed by four-long non-coding RNA (lncRNA) signature and clinicopathologic factors. The four-lncRNA signature was an independent prognostic factor after adjusting age, gender, WHO grades, and 1p19q status in four independent datasets.

Furthermore, the univariate and multivariate Cox regression analyses were conducted to evaluate the prognostic independence of the four-lncRNA risk signature in four independent datasets. As shown in Figure 5, after the clinical factors were adjusted, including age, gender, WHO grades, and 1p/19q status, the four-lncRNA signature was an independent prognostic factor in IDH mutant glioma patients in CGGA RNAseq dataset, TCGA dataset, GSE dataset, and CGGA microarray dataset. These results confirmed the independent prognostic value of the risk score.

### Functional Analysis of Four-Long Non-coding RNA Signature

**Figure 6:**
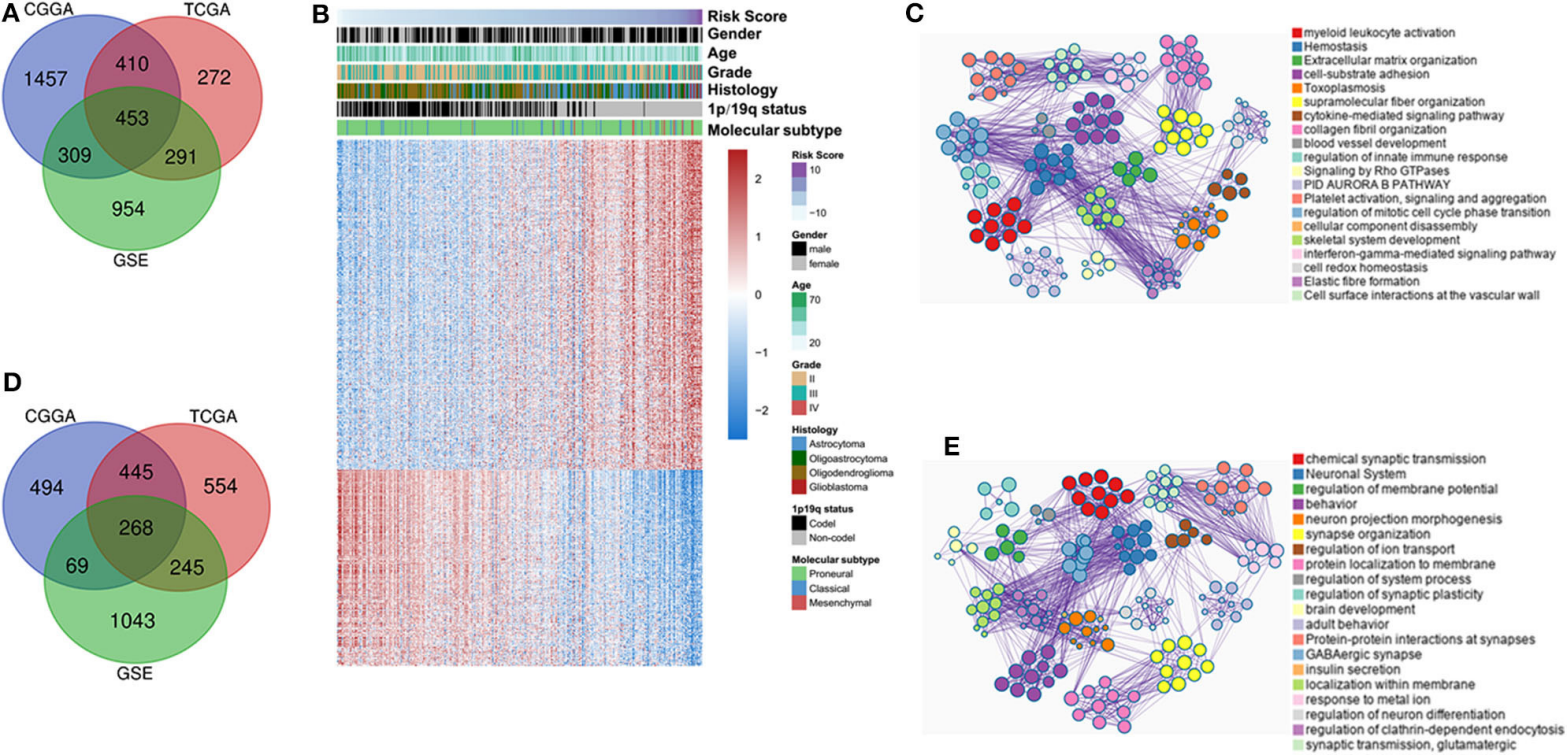
Functional and pathway analyses of four-long non-coding RNA (lncRNA) risk signature in isocitrate dehydrogenase (IDH) mutant glioma. Venn diagram shows the overlapped positive/negative genes of four-lncRNA risk signature in three datasets **(A,D)**. The landscape of clinical and molecular features in associations with four-lncRNA risk score **(B)**. The biological function significantly associated with positive **(C)** and negative genes **(E)**.

To investigate the biological functions of IDH mutant gliomas correlated with four-lncRNA risk signature, we screened co-expressed genes using Pearson correlation analysis (Pearson |r| > 0.4) in CGGA RNAseq, TCGA, and GSE16011 datasets. We found that 453 genes were significantly upregulated (Figure 6A) and 268 genes were significantly downregulated (Figure 6D) in the three datasets. IDH mutant glioma samples were arranged in order of increasing risk factor (Figure 6B). We found that patients in the high-risk group have to 1p/19q intact, WHO II grade and WHO III grade, and classical and mesenchymal TCGA subtypes. The GO and KEGG analyses depicted that the upregulated genes were strongly correlated with the immune-related, cellular component-related, and metabolism-related functions, such as myeloid leukocyte activation, immune response, and extracellular matrix organization (Figure 6C). Meanwhile, the downregulated genes were more involved in normal biological progress, such as chemical synaptic transmission and neuronal system (Figure 6E).

## Discussion

Even with numerous attempts to explore the new molecular and clinical markers to improve the accuracy of diagnosis and prognosis in glioma, the IDH mutant is the most robust and widely applied in clinical practices (10). IDH mutant has been found to be an inciting genetic alteration in gliomagenesis and has profound implications in tumor progression and clinical outcome (11). LncRNA genes are functionally defined as transcripts >200 nucleotides in length with no appreciable peptide products. Recent researches have reported that lncRNA exceeded a more exquisitely regulation function than mRNA in many specific organs (12). The transcriptome profile, genetic alteration characteristics, and methylation data were widely used to stratify the patients in clinical practices (13). However, the alteration of the lncRNA profile in IDH mutant glioma patients remains largely unknown.

Recently, numerous studies reported that lncRNAs emerged as regulators of human carcinogenesis by affecting the expression of key tumor suppressor genes and oncogenes. By analyzing the transcriptomics data from 775 IDH mutant glioma patients, we found that 21 lncRNAs (62.6% of 33 lncRNAs) were upregulated and 12 lncRNAs were downregulated, which suggested that increased lncRNAs play a more important role in IDH mutant glioma. The univariate Cox regression method was further performed to screen the lncRNAs, which were significantly associated with OS in IDH mutant glioma samples in three independent datasets. Finally, four lncRNAs (JAG1, PVT1, H19, and HAR1A) with the independent prognostic value were selected. Among the four lncRNAs, JAG1, PVT1, and H19 were risk-associated genes, while HAR1A was a protective gene.

JAG1 (Jagged Canonical Notch Ligand 1) is located on chromosome 20p12.2 and composed of 26 exons. Notch signaling system, which plays important roles in human development, tissue homeostasis, and disease, is a highly conserved and well-characterized pathway. JAG1 is one of five canonical ligands in Notch signaling pathway expressed by mammalian cells (14, 15). Jag1/Notch axis participated in tumorigenesis and in promoting the progression of colorectal cancer (16), ovarian cancer (17), and cancer stem cell (18). Meanwhile, multiple associations between Jag1/Notch signaling and other oncogenic factors in regulating tumor microenvironment were reported, including interleukins, TGF-β, and the Hippo, Wnt/β-catenin, and NF-κB signaling pathways (19–22). Circular RNAs (circRNAs) are covalently closed loop-like structure with no 5′ to 3′ polarity (23). Plasmacytoma variant translocation 1 (PVT1) gene is encoded by chromosome band 8q24.21 and has both circRNAs and linear lncRNA isoforms (24). The genomic amplification, rearrangements, and increased PVT1 expression exhibited oncogenic and epigenetic regulation effects on breast cancer (25), prostate cancer (26), and acute myeloid leukemia (27). Meanwhile, c-Myc and PVT1 were co-amplified in many cancers (28). In addition, PVT1 was involved in the differentiation of immune cells under physiological and pathological conditions (29, 30). H19 Imprinted Maternally Expressed Transcript (H19), a 2.3-kb lncRNA encoded by chromosome 11p15.5, is associated with the regulation of stem cell differentiation (31). The miR-675-5p, derived from H19 (32), is crucial in downregulated ubiquitin ligase E3 family and subsequently influenced the stability and function of the epidermal growth factor receptor (EGFR) and c-Met (33). Meanwhile, Chao Cui et al. found that H19 plays an important role in the promotion of hypoxia/oxygenation (h/R) injury and apoptosis by induction of autophagy via the inhibiting PI3K–Akt–mTOR pathway in hepatocellular cancer (34). Guan et al. confirmed that H19 is not only overexpressed in glioma tissue and cells, but its expression was also associated with the progression of glioma (35). The investigations performed on H19 indicated that H19 acts as an oncogene in tumors and is associated with tumorigenic phenotypes (36). Highly Accelerated Region 1A (HAR1A), a rapidly evolving cis-antisense segment on chromosome 20q13.33, is mainly expressed in the human brain (37, 38). Previous studies reported that the HAR1A expression levels were reduced in tissue specimens coinfected with hepatocellular carcinoma (HCC) and that low expression of HAR1A in patients with HCC was significantly associated with advanced histological grade and TNM stage. The HAR1A was a favorable prognostic marker for patients with HCC (37). Moreover, consistent with our findings, Zou et al. demonstrated that diffuse glioma patients with lower expression of HAR1A had an unfavorable outcome (39). HAR1A could be potentially used as a prognostic biomarker for HCC and glioma.

The lncRNAs can affect the expression of their flanking coding genes and be functionally related to neighboring coding genes (40). Our analysis indicated that JAG1, PVT1, H19, and HAR1A were aberrantly expressed in IDH mutant gliomas and were core lncRNA genes in regulating the expression of protein-encoding gene in IDH mutant gliomas.

Risk score method is widely applied in clinical studies (41). Here, taking consideration of the expression level and the prognostic contribution of lncRNAs, we established a four-lncRNA risk signature to stratify IDH mutant patients. By applying the risk signature, IDH mutant patients could be divided into the low-risk group and high-risk group. The risk signature exhibited its prognostic value in CGGA RNAseq, TCGA, GSE16011, and CGGA microarray datasets. Patients in the low-risk group significantly had a longer survival than those in the high-risk group. In addition, we found that the four-lncRNA risk signature can also identify high-risk patients in age ≤ 40 and age > 40; grade prognostic prediction power of four-lncRNA risk signature was not decreased in molecular or clinical factor subgroups of IDH mutant glioma. This result emphasized that the mechanism of glioma initiation and progression in low-risk patients and high-risk patients has great distinction. Meanwhile, the univariate and multivariate Cox regression models showed that the four-lncRNA risk signature was an independent prognostic factor.

Moreover, the GO and KEGG pathway analyses revealed that the four-lncRNA signature was positively correlated with leukocyte activation, regulation of innate immune response, and regulation of the Rho GTPase signaling. The risk score was negatively correlated with chemical synaptic transmission, neuronal system, behavior, and regulation of iron transport. The biological functional differences partially interpreted the survival differences between low-risk and high-risk group patients. The abnormal expression of lncRNA genes in the high-risk group could probably influence the outcome of IDH mutant patients by disturbing immune environment and cancer metabolism.

Targeting lncRNAs in cancer is a promising novel approach to cancer treatment. It is well-known that Notch signaling plays a key role in the occurrence, progression, and recurrence of cancer (42). As a novel candidate for blocking Notch signaling, JAG1 had unique characteristics in modifying Notch signal. MicroRNA-26b and microRNA-377-3p could suppress tumor development and invasion through sponging JAG1/Notch signaling (43, 44). The role of PVT1 as a cancer biomarker is widely accepted (45). It has been demonstrated that PVT1 was quantifiable in cancer cells and in serum from cancer patients (46, 47). Knockdown of PVT1 inhibited cell proliferation and arrested cell cycle at G1 stage via recruiting EZH2 (zeste homolog 2) and regulating p15 and p16 epigenetically (28). Moreover, some new insights on the role of PVT1 in drug response are emerging, including the elevated expression of PVT1 in the vincristine-resistant AMU-ML2 DLBCL line (48). Recently, the research performed by Li et al. confirmed that the downregulated expression of H19 increased the efficacy of temozolomide (TMZ) chemotherapy in glioblastoma multiforme (GBM) cells (49). Guan et al. indicated that the downregulation of H19 resulted in Wnt/β-catenin pathway inactivation and thereby inhibited glioma cell proliferation, invasion, and migration (35). Zou et al. found that glioma patients with high HAR1A expression could be benefit more from chemotherapy and radiotherapy (39). Therefore, the lncRNAs are potential therapeutic targets because of the various biological functions in cancers. In addition, the combination of TMZ chemotherapy and lncRNA inhibitor might show synergistic effects in increasing the chances to cure or maintain a long-term remission for glioma patients.

The four-lncRNA risk signature could potentially serve as a prognostic marker for IDH mutant glioma. However, this research has a few limitations. Our study was designed on the basis of retrospective analysis. A large prospective study with large sample size is required to assess the risk score model in future.

To sum up, we established a four-lncRNA risk signature that could predict the survival for IDH mutant glioma patients across multi-platform and multi-population. Our findings indicated that the immune environment and metabolism regulation were more chaotic in IDH mutant glioma patients with higher risk score, and patients in that group may suffer from worse outcome. The combination of lncRNAs was more objective and robust in evaluating the prognosis for IDH mutant glioma patients. The four-lncRNA risk signature could potentially serve as a prognostic score and therapeutic target for IDH mutant glioma patients in the future.

## Data Availability Statement

The original contributions presented in the study are included in the article/Supplementary Material, further inquiries can be directed to the corresponding author.

## Author Contributions

YC: conception, analysis, and design. YG and HC: acquisition of data. FM and HC: reviewed submitted version of manuscript.

## Conflict of Interest

The authors declare that the research was conducted in the absence of any commercial or financial relationships that could be construed as a potential conflict of interest.
