# Supplementary material for: Long Non-coding RNA Expression Profiling Identifies a Four-Long Non-coding RNA Prognostic Signature for Isocitrate Dehydrogenase Mutant Glioma
Source: Front Neurol. 2020 Nov 20;11:573264. doi: 10.3389/fneur.2020.573264 (PMC7714930; doi:10.3389/fneur.2020.573264)
Supplement: Supplementary file 1 [file Table_1.DOCX]

| TUG1 |
| --- |
| LINC00574 |
| DANCR |
| SNHG8 |
| LINC00471 |
| HAR1A |
| SNHG6 |
| HCG18 |
| LINC00032 |
| GRIP2 |
| FAM66C |
| DGCR5 |
| SNHG7 |
| DKFZP434H168 |
| ZNF154 |
| JRK |
| SMCR5 |
| ZNF33B |
| C8orf31 |
| C1orf220 |
| C10orf25 |
| SNHG11 |
| NEAT1 |
| MIR155HG |
| PVT1 |
| H19 |
| NAPSB |
| KIAA0040 |
| MIR22HG |
| LINC00092 |
| OPLAH |
| JAG1 |
| HYMAI |

Table S1. The list of deferentially expressed lncRNA genes
